# Memory complaints and quality of life in a patient with mild cognitive impairment

**DOI:** 10.1192/j.eurpsy.2023.1984

**Published:** 2023-07-19

**Authors:** M. P. Pando Fernández, M. A. Andro Vidal, M. Calvo Valcarcel, P. Martinez Gimeno, M. Queipo de Llano de la Viuda, G. Guerra Valera, A. A. Gonzaga Ramírez, C. De Andrés Lobo, T. Jimenez Aparicio, C. Vilella Martin, M. Fernández Lozano, B. Rodríguez Rodríguez, M. J. Mateos Sexmero, N. Navarro Barriga

**Affiliations:** Hospital clínico universitario de Valladolid, Valladolid, Spain

## Abstract

**Introduction:**

Subjective memory complaints remain a relevant aspect to be considered in patients with mild cognitive impairment. Likewise, their association with depressive symptoms, quality of life and cognitive performance is also an objective to be studied in such patients.

**Objectives:**

Our clinical case represents just one opportunity to study how memory complaints are related to depressive states and how they affect the quality of life of patients with mild cognitive impairment.

**Methods:**

We conducted a bibliographical review by searching for articles in Pubmed.

**Results:**

PERSONAL HISTORY: Male, 73 years old, separated, residing alone in Valladolid. He has home help, a person comes to help him with the household chores. Little social and family circle.

**History in Mental Health:**

He has a history of an admission in 2013 to this Short Hospitalization Unit for ethanol detoxification. Since then, he has been followed up in the Mental Health Unit. According to the reports, he has been diagnosed with depressive disorder and cluster B personality disorder.

Current psychopharmacological treatment: diazepam, olanzapine, duloxetine 60 mg, quetiapine.

Toxic habits: history of chronic ethanol consumption. Smoker. He denies other toxic habits.

**Current Episode:**

The patient presents a worsening of his mood of 15 days of evolution, coinciding with a voluntary decrease of his psychopharmacological treatment that the patient has carried out on his own. He walks with the aid of a crutch. Hypomimic facies. Slowed language, circumstantial, with speech focused on current discomfort.

On assessment, he reports initial improvement after reducing his medication, but in recent days he has experienced a decrease in initiative accompanied by feelings of emptiness, sadness and loneliness. He refers to memory complaints for which he is awaiting evaluation by Neurology. The patient explains that at other times in his life he has presented self-harming ideas that he has been controlling. At this time he expresses desire for improvement and adequate future plans, and accepts plans to attend a memory workshop. He also reports visual hallucinations with no affective repercussions and preserved judgment of reality.

**Therapeutic Plan:**

Treatment adjustment: Duloxetine 60 mg, 2cp/day. The patient is recommended to lead an active lifestyle and attend a day center or memory workshop.

**Conclusions:**

In numerous patients with mild cognitive impairment, we have observed that memory complaints are closely related to depressive symptoms and to the patient’s functioning in daily life.

In one study memory complaints were a negative predictor of quality of life in these patients.

Therefore, in addition to considering the importance of treating depressive symptoms, it is also important to address quality of life in patients with mild cognitive impairment.

**Disclosure of Interest:**

None Declared

